# Patient-Clinician Communication in the Appalachian Region: A Scoping Review

**DOI:** 10.13023/jah.0701.06

**Published:** 2025-05-01

**Authors:** Kawther Al Ksir, Hadii M. Mamudu, Chidiebube J. Ugwu, Emily K. Flores, Tracy Fasolino, Holly Wei, Rick L. Wallace, Florence M. Weierbach

**Affiliations:** East Tennessee State University; East Tennessee State University; East Tennessee State University; East Tennessee State University

**Keywords:** Appalachia, cardiovascular disease, electronic health records, health communication, health disparities, rural health care

## Abstract

**Introduction:**

Effective communication between patients and clinicians is a critical component of quality health care, influencing disease prevention, management, and outcomes. In regions with unique socioeconomic and geographic challenges, communication barriers can further exacerbate health disparities. Understanding the factors that facilitate or hinder patient-clinician communication is essential for developing targeted interventions that improve health care delivery.

**Purpose:**

This review explores the existing literature on patient-clinician communication concerning cardiovascular disease (CVD) in the Appalachian Region with the aim to understand existing gaps and interventions.

**Methods:**

PubMed and Web of Science databases were utilized to conduct a systematic search. The Population, Concept, and Context (PCC) framework guided the inclusion and exclusion criteria, focusing on Appalachian residents and CVD. The selected studies were assessed based on predefined criteria, leading to the inclusion of eight relevant articles. Data analysis was conducted to identify themes and interventions related to patient-clinician communication in the context of CVD.

**Results:**

This review examined interventions emphasizing electronic health records (EHRs), patient engagement, clinician availability, and contextual factors affecting communication. While EHR-based initiatives showed promise in closing preventive care gaps, challenges persisted in addressing patient perspectives and fostering interprofessional collaboration.

**Implications:**

Addressing communication barriers requires tailored strategies that consider patient engagement, clinician availability, and contextual factors, particularly in underserved regions such as Appalachia. Future efforts should prioritize interprofessional collaboration and patient-centered care to enhance equitable cardiovascular health outcomes among diverse populations, including those facing geographic and socioeconomic challenges in Appalachia.

## INTRODUCTION

The impact of cardiovascular disease (CVD) is not equitable with disparities present in both incidence and prevalence throughout the United States (U.S.).^1^ CVD includes conditions such as congenital heart and coronary artery disease (CAD), stroke, myocardial infarction, and cardiac rhythm disorders with hypertension as both a unique disease state and an underlying risk factor for other CVDs.^2^–^4^ Collectively, cardiac conditions are generally the number one cause of morbidity and mortality globally^1^ and in the U.S. regardless of sex, race/ethnicity, and economic strata.^2^ In 2020, the financial impact of cardiovascular morbidity and mortality in the U.S. reached $230 billion in annual economic-attributable costs due to healthcare expenditures and lost economic output.^2^,^3^ Across the U.S., differences in CVD exist, with the Appalachia Region experiencing a higher impact from CVD than the national average.^6^ Within Appalachia, CVD mortality is 17% higher than the national rate, with rural area mortality even higher at 34%.^6^ As such, it is paramount that clinicians across the U.S. address disparities and inequities in CVD with a focus on high-burdened regions such as Appalachia.^2^

Patient-clinician relationships include establishing mutual healthcare goals and effective communication.^7^ Clinicians have responsibility for ongoing assessment, medication management, education, and ongoing support,^7^ while the patient has responsibility for clear communication addressing how they feel and what they do to be healthy. The clinician and the patient have needs, values, beliefs, and emotions that influence how they interpret and convey messages. Considering this, an initial step in CVD management should include meaningful communication between clinicians and patients to achieve a better health outcome. To achieve maximum health of Appalachian residents, worthwhile communication must occur with an awareness of the external factors that may influence communication (such as the environment), including technology and healthcare infrastructure. Collaboration, such as team-based health care, is a strategy that may be employed to assist in effective communication between clinicians and patients. The emphasis of team-based health care places the patient in the center of the care team, frequently referred to as patient-centered care.^9^ Engaging patients and their caregivers at the center of care by using a team-based upstream approach is an area that may be part of the solution for optimizing CVD management. This combination of relationship and communication factors within the context of place influence how patients and clinicians not only perceive information but extends to how health care communication occurs.^8^

The existing evidence demonstrates that patient-clinician communication and relationships that focus on CVD management and outcomes are related to higher medication adherence, lower healthcare expenditure, and higher consumer satisfaction.^10^ This scoping review aims to map the body of the extant literature on patient-clinician communication about CVD to inform future research involving socioeconomically disadvantaged and medically underserved communities in Appalachia and beyond.

## METHODS

### Sample Search Strategy

PubMed and Web of Science were initially searched for eligible articles. The search terms selected from the pre-established MeSH terms included “Appalachian Region” and “physician-patient relations OR communication AND cardiovascular disease.” The PCC (Population, Concept, and Context) framework was used to guide this research strategy. The population was chosen to be Appalachian residents (all age groups, all genders, all races and ethnicities). While the concept was defined as CVD, the context was defined as patient-clinician communication.

### Study Selection Process

**Figure 1:**
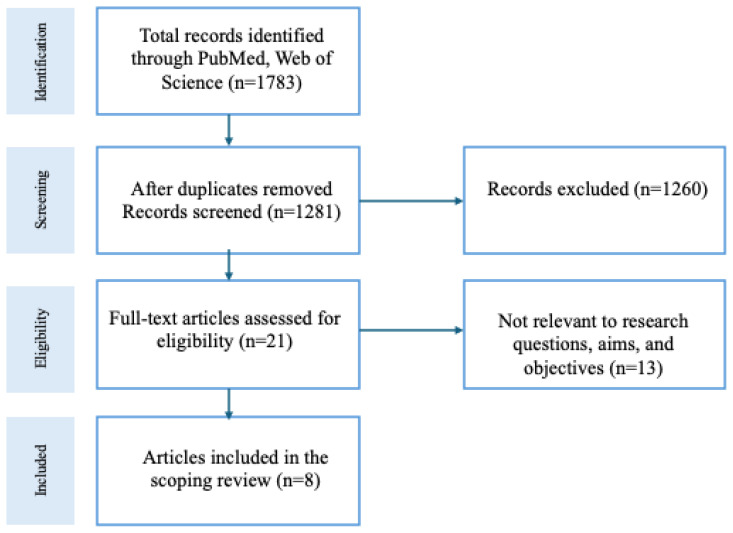
Study Selection Process

The primary author examined the text words in the title and abstract of the retrieved papers, as well as the MeSH terms used to describe the articles. Articles written prior to 1990, those conducted outside of the Appalachian Region, reports, editorials, commentaries, grey literature, abstracts, posters, conference proceedings, narrative reviews, and finally, articles written in a language other than English were excluded. Four screeners located and evaluated full-text articles that are potentially relevant for inclusion. In case of uncertainties, a second reviewer was consulted to resolve the differences. Fig. 1 outlines the processes used in the review.

The preliminary literature search yielded 1783 articles (Pubmed: 1646, Web of Science: 137). Of those, 502 were duplicates and 1260 did not meet the inclusion criteria **(**Fig. 1). After reading the full-text articles, eight records were included in this scoping review. Study characteristics, including purpose, methods, findings, and implications, were extracted from each article and themes identified. Studies were grouped by design for reporting.

## RESULTS

All publications employed a quantitative methodology, with four descriptive^11^–^14^ and the remainder including interventions.^15^–^18^ Study characteristics are summarized in Table 1 for descriptive studies and Table 2 for intervention studies.

Four (4) major themes were identified related to patient-clinician communication about CVD in these Appalachian populations: (1) interventions promoting communication, (2) patient engagement, (3) clinician availability, and (4) contextual factors.

### Theme 1: Interventions Promoting Communication

This theme addresses technology and human interaction interventions that promote communication with the main purpose of improving the health of patients diagnosed with CVD. All the interventions utilized technology, with some focusing on patient-clinician communication while others employed a reminder system for clinicians to address CVD management and treatment with the next patient interaction. These technologies included electronic health records (EHR), emails, and undesignated patient portals.

Technology that serves as a digital repository of a patient's medical record allows the clinician rapid access to a patient's health information. Within the EHR mechanisms, provider reminders, including the ability for clinician interactions, are available to assist with the coordination of care. Other technologies, such as emails and patient portals, focus on the patient and provide them access and control over their health information. Non-EHR technologies are not as comprehensive and are frequently viewed as personal health records. Details of EHR and additional technology provide information that advances knowledge of patient-clinician communication.

One EHR intervention evaluated patients’ response patterns to receive notifications through a personalized health record (PHR) embedded within the EHR regarding guideline-recommended services for chronic medical conditions.^16^ This study compared the number of reminder messages sent to the clinician; the results indicated clinician participants increased their access to PHR or care by 12% (from 61% to 73%) with two reminder messages. However, there were no improvements in PHR access or care with subsequent increases in reminder messages, indicating that there might be a limited effect on reminder messages.^16^ This study revealed that gaps in care that did not require a clinician visit, such as lab tests, had a higher closure rate. This trend can be attributed to several factors.^16^ First, these gaps are easier to close, as tasks like completing a lab test often do not require scheduling an appointment, making them more convenient for patients to address. Second, fewer notification cycles are needed for simpler tasks like blood tests or documentation compared to those requiring an office visit, suggesting that patients respond more readily to reminders for less complex actions. In contrast, gaps that require a clinician visit, such as mammograms or scheduled appointments, tend to have lower closure rates due to the additional planning and time commitment involved. The study also found that a significant number of patients accessed the PHR or received care after the first notification, indicating that reminders are particularly effective for straightforward tasks.

A separate intervention study compared two interventions, the EHR reminder (RO) and the clinician training and reminder (R+T) for patients’ blood pressure care.^17^ This intervention study tested clinicians' reminder only versus clinicians' reminder plus training on communication skills (R+T). The findings showed that patients’ blood pressure improved overall. The patients in the R+T group had a significant decrease in blood pressure compared to other groups.^17^ While these findings are inconclusive, the results may suggest that reminders alone may not improve patients’ self- care for their blood pressure.

Another intervention was a randomized controlled trial examining the effectiveness of a nurse coach approach to improve patient and clinician communications.^15^ Coaching included teaching patients how to use the identified internet portal to interact with their clinicians. In addition to the use of the portal, nurse coaching included requesting prescription refills, scheduling appointments and referrals, and reviewing medical records. The participants in the intervention group reported higher satisfaction with their visits to the primary care provider, while the control group reported that their primary care providers offered advice on their health or a referral to a specialist. The last intervention was a prospective pre-post implementation study examining invasive procedure consent with low English proficiency (LEP) individuals.^18^ The study included two groups of participants with LEP. The group with 24-hour access to professional interpreters via phone demonstrated a better understanding of key elements of the consent.

The identified EHR interventions in this review showed various degrees of effectiveness. However, statistical significance was not reached. Some effects were present, with the first study using EHR as the intervention to teach patients about guideline-recommended services on chronic medical condition management. Inconclusive findings were present in the second study that compared two different interventions using the EHR. Findings were positive in the randomized controlled trial for the intervention utilizing a nurse and a patient portal.^19^

### Theme 2: Patient Engagement

This theme addresses communication and the relationship between clinicians and patients. Communication is the cornerstone of patient engagement with a shared and mutual exchange of information. Inconsistencies were present in a clinician study focusing on patient-clinician relationships.^13^ This study addressed clinicians engaging patients in lifestyle choices with no clear mention of the patient’s disease process.^13^ Patient engagement extends beyond the patient-clinician relationships to also include coordination among clinicians. Another study demonstrated the importance of relationships among team members. The same study addressed relationships between team members, the team, and the patient.^20^ When addressing relationships, the degree of coordination among the team determines the team effectiveness and ease of communication present with the patient.^20^

### Theme 3: Clinician Availability

This theme addresses continuity of care and its impact on CVD detection rates and outcomes. One study demonstrates that CVD detection rates vary based on continuity of care.^11^ Patients who maintained continuity of care with a consistent clinician exhibited better detection rates compared to those who underwent a change in clinicians during the course of their medical care.^11^

Insufficient availability of clinicians within vulnerable demographic groups may contribute to the disparity in health outcomes linked to CVD. Specifically, black and elderly populations lacking continuity of care are at a higher risk of developing hypertension and its associated complications.^11^ Available literature focuses on black patients’ medication adherence and clinician communication.^11^ Clinicians of black patients were significantly more active in advising and counseling patients about hypertension care and medication adherence.^11^ Black patients reported more knowledge or awareness of the importance of blood pressure control.^17^ However, when compared to nonblack patients, no differences were present in medication adherence.^17^

Continuity of care involves relationships between clinicians and patients and may be influenced by the availability of data that address this dynamic. While continuity of care is important and appears to lead to better health outcomes, appreciating the dynamic between the clinician and patient is important and needs available data on all study participants to have a comprehensive picture addressing continuity of care.^11^,^17^ In this theme, the focus is on continuity of care and clinical outcomes, not on clinician characteristics. While patient characteristics are accounted for using a population lens, moving forward, it is important to consider how clinician characteristics contribute to continuity of care and clinical outcomes.

### Theme 4: Contextual Factors

This theme focuses primarily on patient attributes, including language, health literacy, socioeconomic factors, and duration of disease/illness. Contextual factors, while traditionally viewed at an individual level, transcend how communication happens and where it occurs.

The Appalachian Region has multiple vulnerable populations, based on socioeconomic and cultural factors. Limited financial resources are present throughout the region, with residents being predominantly English-speaking. However, with the increase in the Hispanic population^5^ with limited English proficiency (LEP), healthcare systems are challenged to provide interpretation services. These services vary from in-person interpreters, family members, and technology assisted interpretation,^18^ including the availability of telephone access with interpreter services using artificial intelligence (AI).

Language and health literacy have been linked together for patients with LEP. A study using telephone-based professional interpreters for patients, predominantly Spanish-speaking with LEP, was noted to have an improved understanding of scheduled medical procedures.^18^ However, while telephone-based professional interpreters may be available, they may not be accepted and utilized by those who need the service.^18^

Literature suggests that adequate communication presupposes the bridging of health barriers and health literacy.^19^ Furthermore, concern is present that links communication and health literacy to social determinants of health (SDOH) including economics, education, healthcare access, and geography. Addressing contextual factors associated with communication needs to include place-based and patients’ personal SDOH. One study demonstrates that patients in outpatient clinics are more likely to take charge and coordinate with their clinicians to gain some control over their own health.^19^ Personal attributes, such as depression, may impact patient-clinician communication and subsequent health behavior.^9^

## DISCUSSION

The findings of this review did not encompass discussions on disadvantaged and underserved populations or models of care designed to tackle the existing disparities and inequities in CVD. The four (4) major themes are (1) interventions promoting communication, (2) patient engagement, (3) clinician availability, and (4) contextual factors. Three themes address communication with the interventions promoting communication, clinician availability, and engagement between the patient and the clinician.^15^–^18^ The themes addressing patient-clinician communication heavily relied on technology.^15^–^18^ The primary technology focus of the three themes was on clinicians utilizing electronic prompts to address treatment and ensure adherence to the care plan.^15^–^17^ Technology use for patient-clinician communication was primarily centered on EHR portals and other electronic methods of communication.^15^–^17^ While there were technology-based interventions addressing communication, the inconsistency and varied implementation resulted in outcomes that lacked practical applicability in real-world practice. As AI advances, the use of it will increase, and as such, it will be important to address whether its use is seen as a facilitator or barrier for communication between clinicians and patients. The fourth theme, contextual factors, focuses on the patient.

It is essential to recognize and validate the significance of human connections fostered through verbal communication. The theme of contextual factors addresses the importance of communication with patient-centered care. However, it is challenging to discern the relationship between the clinician and the patient within the theme. Instead, communication interventions utilized technological interventions with clinicians in their delivery of care with minimal attention to contextual factors at the patient or environmental level. Environmental contextual factors may be viewed through the lens of social drivers, which may be seen as parallel to SDOH at the individual level. In Appalachia, these factors include limited internet and cell phone access along with transportation, geography, and provider access, which could create barriers to communication between provider and patient.^5^ Healthcare provider decisions to use technology to replace verbal communication have the potential to remove the patient from the relationship and potentially place the patient in a dependent downstream position. Collectively, addressing context at the individual and environmental level may assist in defining the importance of including technology as context to address the use of AI and other technology-based communication.

Further, given the limited availability of data on the percentage of CVD patients in the reviewed studies, this gap should be acknowledged as a limitation when interpreting the findings. Without a clear understanding of the proportion of CVD patients represented, it becomes challenging to assess the generalizability and applicability of the identified communication barriers and interventions. Future research should aim to include more comprehensive patient demographic data to strengthen the validity of conclusions and ensure that findings accurately reflect the broader CVD population, particularly in underserved regions like Appalachia.

## IMPLICATIONS

Multiple factors contribute to health outcomes with disadvantaged and underserved populations with known health disparities. SDOH have gained stature in health equity research with emphasis placed on education, healthcare access, and financial resources. However, the SDOH component addressing relationships does not have the same level of stature in health equity research. Future research with disadvantaged and underserved populations must include a mechanism to address communication and relationships between clinicians and patients. Addressing relationships through the lens of SDOH is novel.

Multiple research methods at both the individual and population levels allow for the inclusion of addressing relationships. At the individual level for both the clinician and patient, including a self-report of satisfaction with the use, efficiency, and effectiveness of communication methods. Self-reports addressing communication may be part of the demographic information, with either open-ended questions, yes/no, or Likert scale options. A follow-up question addressing relationships between the client and patient as it relates to communication provides further information that may be key to addressing health outcomes. At the population level, it is paramount to explore healthcare policies at the local, state, and national level that address communication. Policies may include how HIPAA is operationalized with technology.

Health equity research addresses SDOH, however placing provider patient relationships in the context of communication is an area that needs further study. A mechanism to address all SDOH should be included in all health outcome research. The impact of communication between providers and patients is an area that must be included in health equity research to fully operationalize the concept of patient-centered care. To fully embrace disparities that are manifested through health inequities, it is paramount to assess and use models of care that influence relationships and communication between patients and clinicians. Interprofessional teams promote relationships among clinicians, enabling patients to engage with multiple different clinicians. This approach addresses the diverse needs of patients while also acknowledging and considering the role of each clinician in the healthcare process. Research addressing patient-centered care in the interprofessional team model needs further exploration as this may be a key to unraveling the disparities within disadvantaged and underserved populations.

To achieve CVD-associated health outcomes set in Healthy People 2030 for disadvantaged and underserved populations such as those in the Appalachian Region, patient-centered care must be included in research projects.^20^

## CONCLUSION

In conclusion, this scoping review examines patient-clinician communication regarding CVDs in the Appalachian Region. While findings suggest that while communication is present, the quality remains limited due to several factors. The paucity of research suggests that the amount of communication, along with the lack of a standardized model that clinicians can follow, all lead to not addressing social relationships within the SDOH model.

**SUMMARY BOX**

**What is already known about this topic?**

Effective patient-clinician communication is essential for chronic disease prevention and management, particularly for cardiovascular disease (CVD). Patients in underserved regions, such as Appalachia, face significant barriers to healthcare access, including socioeconomic challenges, limited clinician availability, and technological constraints.

**What is added by this report?**

This scoping review synthesizes existing literature on patient-clinician communication regarding CVD in Appalachia, identifying key themes such as the role of EHRs, clinician availability, and contextual factors influencing communication. While some interventions show promise in bridging preventive care gaps, challenges persist in addressing patient perspectives and fostering interprofessional collaboration. The review highlights critical gaps in data, particularly regarding the proportion of CVD patients affected and the effectiveness of tailored communication strategies.

**What are the implications for future research?**

Future research should focus on developing and evaluating patient-centered communication strategies that address the unique barriers faced by Appalachian communities. This includes expanding the use of EHR-based interventions while ensuring they align with patient needs and preferences. Additionally, studies should incorporate more comprehensive demographic data to enhance generalizability and assess the impact of targeted communication approaches on CVD outcomes. Strengthening interprofessional collaboration and integrating culturally relevant strategies will be essential in improving healthcare delivery and reducing disparities in cardiovascular health.

## Figures and Tables

**Table 1:** Sumary of Descriptive Studies Included in the Review

First Author, Year	Study design	Study Population	Key Findings
Konrad et al., 2005^11^	Longitudinal cohort study from 1986–1990 to assess the effects of clinician-patient racial concordance and continuity of care on hypertension outcomes	4162 patients over 65 years of age Piedmont-area of North Carolina In-home visitation part of Piedmont health survey of the elderly hypertensive patients	Continuity of care with the same clinician is crucial for treatment adherence This importance remains consistent across various factors such as patient income It is equally important regardless of the patient's race The race match between the patient and clinician does not diminish the significance of continuity of care for treatment adherence
Kressin et al., 2007^12^	Survey assessment to examine the effectiveness of utilizing reminders for blood pressure checks, comparing it to clinician training focused on enhancing health communication through verbal reminders	793 White and African American patients From 3 urban tertiary care Department of Veterans Affairs (VA) Medical Centers Survey assessments of certain dimensions subsequent to a primary care clinic visit 58% patients previously diagnosed with hypertension	Black patients received active counseling on hypertension care. Adherence to hypertension care did not show significant variation by race Medication adherence was influenced by factors such as pill-splitting instructions, perceived high blood pressure, and clinician guidance Confidence in medication usage positively correlated with improved adherence Emphasizes the importance of both clinicians and patients prioritizing blood pressure management The goal is to reduce disparities in adherence and control, emphasizing the need for a comprehensive approach
Viera et al., 2011^13^	Cross sectional study to access if clinicians informed patients of their pre-hypertensive status	1008 patients From seven primary care practices within the North Carolina Family Medicine Research Network (NC-FM-RN)	Clinicians may not engage patients in prehypertension discussions due to perceived lack of usefulness in practice Clinicians might focus on counseling patients about lifestyle modifications to reduce hypertension risk without explicitly mentioning prehypertension The possibility exists that clinicians prioritize general hypertension prevention strategies over discussing prehypertension with patients
Kripalani et al., 2016^14^	Prospective cohort study to examine the relationships between social determinants of health and outcomes after hospital discharge	1967 patients From Vanderbilt Inpatient Cohort Study who were admitted for coronary syndromes or heart failure	Lower medication adherence in cardiovascular patients is associated with non-white races Age extremes, lower education levels, and lower income/socioeconomic status may contribute to lower medication adherence in cardiovascular patients, although results have been inconsistent. Medication adherence is likely influenced by psychosocial factors such as health literacy Numeracy, self-efficacy, social support, and depression are additional psychosocial factors that can impact medication adherence in cardiovascular patients

**Table 2:** Summary of Intervention Studies Included in the Review

First Author, Year	Aims	Study Population	Study Design & Intervention	Outcome Measures	Key Findings
Leveille et al., 2009^15^	To test the effectiveness of portal-based coaching to promote patient-primary care chronic condition discussions	241 Patient-portal patients	The study was a randomized, controlled trial based on behavioral change featuring brief, targeted intervention to encourage patients to discuss their screened chronic condition with their clinician at their next visit	Patient report on primary care visit Condition specific screening tools Centers for Disease Control and Prevention Healthy Days Measure	No significant difference in health outcomes, but the intervention group reported a better visit experience with their clinician
Hess et al., 2014^16^	To evaluate patient response patterns and follow-through after receiving an email notification about preventive care gaps for chronic illnesses	584 Patients with high cardiovascular risk	Up to three weekly interval e-mail messages notifying the patient of a preventable care gap specific to their chronic disease diagnosis that needs to be addressed. The following reminder message would be resent if all preventive gaps were not closed in two months. If the patient did not respond after three messages, the next reminder message would be in two months, and the cycle of messages would restart.	Messaging-Cycles Gap-Cycles - Percentage of closed gaps	At the beginning of the study, there were 2,656 unaddressed gaps After the study, 58% were closed Patients continued to respond to messages after a gap had been closed Gaps in care that did not require a clinician visit, such as lab tests, had a higher closure rate.
Kressin et al., 2016^17^	To evaluate the effectiveness of three clinician-focused interventions (with two interventions using EHR reminders) to improve hypertension in white and black veterans	N = 8,866	The study had three arms with site randomizations. There were three interventions: (1) usual care (control), (2) EHR reminder only (RO), (3) EHR reminders and trained clinicians to advise and counsel patients about medication adherence and hypertensive care (R + T).	Patient sociodemographic and clinical characteristics Clinician counseling behaviors Hypertension medication adherence Blood pressure	The R+T and RO arm showed statistically significant improvement in counseling behavior White patients received more counseling than black patients There were no statistical improvements in medication adherence at follow-up All arms saw decreased blood pressure over time Intervention effect; R+T, and RO decreased in DBP compared to control and decreased in the percentage of uncontrolled compared to controlled arm
Lee et al., 2017^18^	Prospective, pre/post intervention implementation study	152 patients in the Intervention Group and 86 in Control Group Hospitalized patients undergoing invasive procedures on the cardiovascular (46.4%), general surgery (33.3%), or orthopedic surgery (20.3%) floors.	Installation of dual-handset interpreter phones at every bedside, enabling 24- hour immediate access to professional interpreters	Primary outcomes: three central informed consent elements, patient- reported understanding of the (1) reasons for and (2) risks of the procedure and (3) having had all questions answered. Consent was considered adequately met when patients were informed about all three elements.	The implementation of a bedside interpreter phone system intervention to increase rapid access to professional interpreters was associated with improvements in patient-reported informed consent for patients with Low English Proficiency (LEP) undergoing invasive procedures and should be considered for all hospitals seeking to improve quality and decrease disparities for patients with LEP
